# Modulation of LPS stimulated NF-kappaB mediated Nitric Oxide production by PKCε and JAK2 in RAW macrophages

**DOI:** 10.1186/1476-9255-4-23

**Published:** 2007-11-24

**Authors:** Edward Jones, Ian M Adcock, Bushra Y Ahmed, Neville A Punchard

**Affiliations:** 1 Division of Science, University of Luton, Luton. UK; 2 Airway Diseases, NHLI, Imperial College London, London. UK; 3 School of Health & Biosciences, University of East London, London. UK

## Abstract

**Background:**

Nuclear factor kappa B (NF-κB) has been shown to play an important role in regulating the expression of many genes involved in cell survival, immunity and in the inflammatory processes. NF-κB activation upregulates inducible nitric oxide synthase leading to enhanced nitric oxide production during an inflammatory response. NF-κB activation is regulated by distinct kinase pathways independent of inhibitor of κB kinase (IKK). Here, we examine the role of protein kinase C isoforms and janus activated kinase 2 (JAK2) activation in NF-κB activation and LPS-stimulated NO production.

**Methods:**

Murine RAW 264.7 macrophages were treated with lipopolysaccharide (LPS), Phorbol 12-myristate 13-acetate (PMA) and a combination of LPS and PMA in the presence or absence of various inhibitors of PKC isoforms and JAK2. Nuclear translocation of the NF-κB p65 subunit, was assessed by Western blot analysis whilst NO levels were assessed by Greiss assay.

**Results:**

LPS-stimulated NO production was attenuated by PMA whilst PMA alone did not affect NO release. These effects were associated with changes in p65 nuclear translocation. The PKCα, β, γ, δ and ζ inhibitor Gö 6983 (Go) had no effect on LPS-induced NO release. In contrast, Bisindolymalemide I (Bis), a PKC α, β_I_, β_II_, γ, δ and ε isoform inhibitors completely inhibited LPS-stimulated NO production without affecting p65 nuclear translocation. Furthermore, a partial inhibitory effect on LPS-induced NO release was seen with the JAK2 inhibitor AG-490 and the p38 MAPK inhibitor SB 203850.

**Conclusion:**

The results further define the role of NF-κB in LPS stimulated NO production in RAW macrophages. The data support a function for PKCε, JAK2 and p38 MAPK in NF-κB activation following p65 nuclear import.

## Introduction

Increasing emphasis is currently placed on the role of the innate immune system in inflammatory responses, in particular those involving macrophages. As in other cells, the transcription factor, NF-κB plays a pivotal role in changes in gene expression during such inflammatory responses. A range of inflammatory stimuli, including endotoxin [1,2] and cytokines [3], produce activation and nuclear translocation of NFκB following rapid degradation and release of IκB.

One of the genes upregulated by NF-κB during an inflammatory response is the inducible nitric oxide synthase (NOS2), that produces nitric oxide (NO), a highly reactive free radical with important second messenger functions involving the mediation of inflammatory events [4]. Increased expression of NOS2 and concomitant NO levels have been reported in several inflammatory diseases, such as Crohn's disease [5], asthma [6] and rheumatoid arthritis [7]. The NOS2 gene promoter contains twenty two putative transcription factor binding elements [8], however, so far only the NF-κB responsive κB element [9] and an interferon-γ-activated site (GAS) [10] have been shown to enhance NOS2 expression.

Macrophages are the primary producers of NO *in vivo *and one of the dominant cell types to display NF-κB activation in inflammatory diseases [10]. Lipopolysaccharide (LPS) stimulates NO production in macrophages. The induction of NOS2 protein expression in response to stimulation with LPS involves the Janus kinase (JAK) family of protein kinases [11]. Furthermore, both protein kinase C (PKC) and Janus Kinase2 (JAK2) [12-14] have been implicated in NF-κB activation. However, although nine isoforms of PKC have been identified in macrophages [15] it is unknown which of these are involved in NF-κB activation.

PKC activation has been identified as an early response in LPS-stimulated macrophages [16] and is essential for the up-regulation of NO production [16,17] However, the function of PKC isoforms involved in upregulation of NO production remains to be determined. Thus PMA, a direct activator of the PKC family of kinases, was used to investigate the role of PKC in LPS-stimulated NO production and NF-κB activation in RAW cells. PMA has been shown to induce a PKC mediated proteasomal-independent pathway of NF-κB nuclear translocation in human intestinal epithelial cells [18].

The present study uses pharmacological tools to indicate a role for PKCε in LPS-stimulated NF-κB-mediated NO release in RAW macrophages. We also implicate a role for JAK2 and p38 MAPK on these effects.

## Methods

### Cell Culture

RAW 264.7 cells (ECACC, Salisbury, UK) were maintained in 25 cm^2 ^flasks in DMEM medium supplemented with 2 mM L-glutamine and 10% v/v FCS, without antibiotics, at 37°C in a humidified atmosphere of 95% air and 5% CO_2_. For Western blotting, cells were grown in 25 cm^2 ^flasks, whilst for the measurement of NO the cells were grown to 95% confluence in 96-well plates and stimulation carried out within these plates. Cells were stimulated by replacing the culture medium with medium containing LPS, LPS with phorbol-12-myristate-13-acetate (PMA) or PMA alone in the presence or absence of various inhibitors.

Inhibitors used were: the PKC inhibitors Gö 6983 (Go) and Bisindolymalemide I (Bis); the JAK2 inhibitor AG-490; the p38 MAP kinase inhibitor SB 203580 (Calbiochem, Nottingham, UK). Bis shows high selectivity for PKC α, β_I_, β_II_, γ, δ and ε isoforms at 20 μm [19] whilst Go inhibits PKC α, β, γ, δ and ζ isoforms at 10 μm [20]. AG-490 was used at 10 mM, a concentration previously shown to inhibit JAK2 [21], and SB 204580 at 10 mM, a concentration previously shown to inhibit the p68 MAP kinase family [22]. For the purpose of specific inhibition of PKC translocation, the following MALY-TAT linked peptides (kindly supplied by Dr M. Lindsay, AstraZeneca, Charnwood, UK) were used: MALYO1 (TAT- RFARKGALRQKNHEVK), MALY1O (TAT-EAVSLKPT), MALY II (TAT-LSETKPAV0) at concentrations previously shown to inhibit translocation of PKC isoforms [23]. For Western blotting cells were incubated for 0, 1, 2, 3 or 5 hours, whilst for the NO assay, cells were incubated for 24 hours.

### Assessment of NF-κB-p65 nuclear translocation by Western blot analysis

RAW 264.7 cells were harvested in ice cold PBS after stimulation with LPS from 0 to 5 h. Cells were then lysed in 70 μl of buffer A (10 mM HEPES pH 7.9, 1.5 mM MgCl_2_, 10 mM KCl, 0.25% v/v noident P-40, 0.5 mM dithiothreitol (DTT), 0.5 mM phenylmethylsulfonyl fluoride (PMSF) in de-ionised water (dH_2_O) for 20 min on ice, to yield the cytoplasmic cellular fraction, as described previously [24]. The samples were microfuged at 12,000 g for 15 sec to pellet the unlysed nuclei and the supernatant (cytoplasmic fraction) was collected. The nuclei were lysed in 15 μl of buffer B (20 mM HEPES pH 7.9, 1.5 mM MgCl_2_, 0.42 M NaCl, 0.5 mM DTT, 25% v/v Glycerol, 0.5 mM PMSF in diH_2_O) for 20 min on ice, and microfuged or centrifuged at 12,000 g for 60 sec to pellet the cellular debris. The supernatant (nuclear fraction) was collected and 60 μl of buffer C (20 mM HEPES pH 7.9, 50 mM KCl, 0.5 mM DTT, 0.2 mM EDTA, 0.5 mM PMSF) was added to it. At this stage the protein concentration of the samples was assessed by BioRad protein assay ™ (Biorad, UK).

Samples (10 μg) were separated by 10% SDS-PAGE and proteins transferred to a nitrocellulose membrane (Amersham-Pharmacia, Amersham, UK) by electroblotting. Equal protein loading was confirmed by Ponceau S staining of the membrane. Non-specific protein binding was blocked by incubation of the membrane in PBS-T + 1% w/v milk overnight at 4°C. Membranes were then washed twice in PBS-T for 5 min before incubation for 1 hour at room temperature (RT) with rabbit anti-p65 antibody (1:4000, Santa Cruz, Wembley, UK). Membranes were then washed twice in phosphate buffered saline (pH 7.4) containing 0.05% v/v Tween 20 (PBS-T) and 1% w/v milk for 5 min followed by an hour incubation at room temperature (RT) with goat anti-rabbit HRP conjugate (1:4000, Dako, UK). All antibodies incubations were carried out in PBS-T containing 1% w/v milk. Membranes were washed three times for 5 min in PBS-T before incubation with ECL substrate (Amersham-Pharmacia, UK), followed by exposure to an autoradiographic film and subsequent semi-quantification of band intensity by densitometry (UVP Ltd, Cambridge, UK).

### Nitrite determination by Greiss assay

NO levels were assessed by nitrite quantification as described previously [25]. Briefly, 90 μl of sample (cell culture medium) was incubated for 5 min in dark at RT with 90 μl of suphanilamide (1% w/v in 4 M HCl). 90 μl of napthylethylenediamine (1% w/v in dH_2_O) was then added and a further 5 min incubation was carried out in dark at RT. Absorbance was read at 540 nm.

All reagents were purchased from Sigma (Poole, UK) unless otherwise stated above.

### Statistical analysis

Data are reported as mean ± SEM. Statistical analysis was performed in Prism 5 (Graph Pad Software, Inc. San Diego, USA) using one way analysis of variance (ANOVA) followed by Tukey's Multiple Comparison Test (TMCT) when ANOVA indicated a statistical significance existed.

## Results

### The effect of LPS and PMA on RAW cell NO production

**Figure 1:**
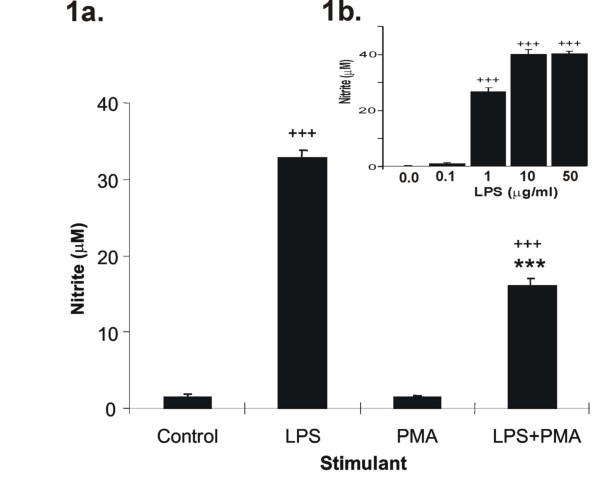
**a. The effect of PMA on LPS stimulated NO production in RAW 264.7 cells**. RAW cells were stimulated for 24 hr with either vehicle alone (control), 1 μg/ml LPS, 50 ng/ml PMA or 1 μg/ml LPS with 50 ng/ml PMA. NO levels were assessed by Greiss assay. LPS alone significantly increased NO production whereas PMA alone had no effect. PMA significantly inhibited LPS -stimulated NO production. **Figure 1b **(inset) displays the concentration-response curve for LPS-stimulated NO production over 24 hrs. Results are expressed as mean ± SEM; ^+++^p < 0.001 vs control, ***p < 0.001 vs LPs-stimulated; n = 6 for the effects of PMA and n = 9 for the concentration-response curve.

Constitutive NO production by RAW 264.7 macrophages (Fig. 1) was at the lower limit of the sensitivity of the assay (3 μM,). LPS induced a concentration-dependent increase in NO production with a maximum response at 50 μg/ml at 24 h (Figure 1b.). For subsequent experiments, LPS (1 μg/ml) was chosen for its ability to stimulate high levels of NO production whilst not possessing the significant (p < 0.001) toxicity seen with 10 and 50 μg/ml (38% and 51% reduction in viability respectively).

LPS stimulation induced NO production with nitrite levels peaking at 33 μM (p < 0.001, Fig. 1), in agreement with the results of others [26,27]. In contrast, PMA alone (0.5–500 ng/ml) had no effect on NO production but significantly attenuated LPS-induced NO production by ~50% (Fig. 1) even at concentrations (50 ng/ml) previously shown to activate NF-κB.

### The effect of LPS on NF-κB activation

**Figure 2:**
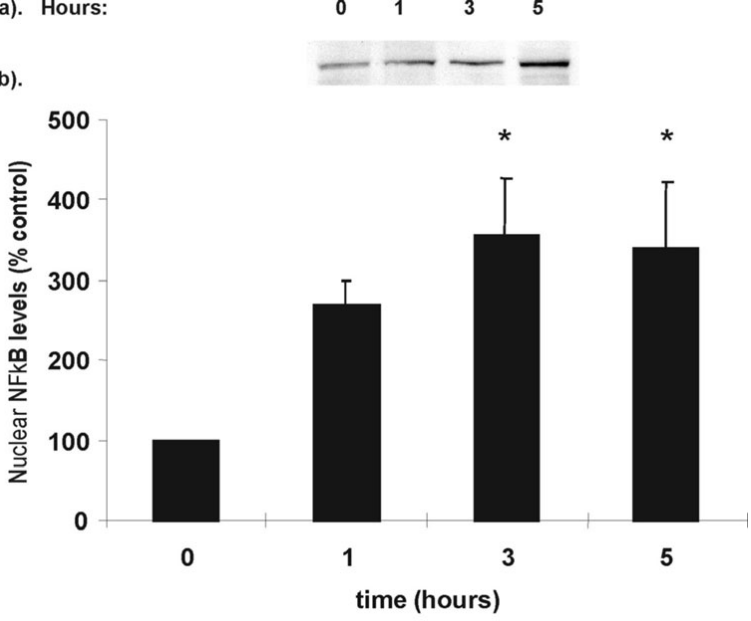
**NF-κB activation in LPS stimulated RAW 264.7 cells**. RAW cells were stimulated with LPS (1 μg/ml) for between 0–5 hours. Cells were harvested and nuclear NF-κB-p65 levels were assessed by Western blotting. (a) Representative Western blot of p65 expression. Equal amounts of nuclear proteins are loaded onto each lane. (b) Graphical representation of % increase in p65 nuclear localisation shown in (a) above. Data is presented as mean ± sem, *p < 0.05, n = 6 independent measurements.

**Figure 3:**
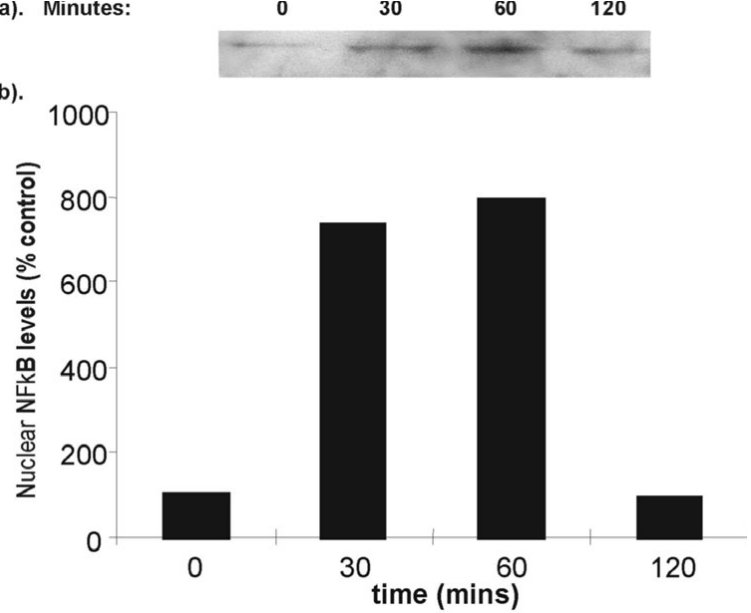
**NF-κB activation in LPS and PMA stimulated RAW 264.7 cells**. RAW cells were stimulated with LPS (1 μg/ml) and PMA (50 ng/ml) for between 0–120 minutes. Cells were harvested and nuclear NF-κB-p65 levels were assessed by Western blotting. (a) Representative Western blot of p65 expression. Equal amounts of nuclear proteins were loaded onto each lane. (b) Graphical representation of % increase in p65 nuclear localisation shown in (a) above. Data is presented as mean of 2 independent experiments.

LPS (1 μg/ml) induced a significant 4-fold induction of p65 nuclear translocation which was maintained for up to 5 hours (p < 0.05, Fig. 2). PMA alone also significantly induced NF-κB activation (data not shown), however, combined effect of PMA and LPS-stimulated NF-κB showed an 8-fold increase within 30 minutes PMA reduced the duration of LPS-stimulated p65 nuclear translocation from > 5 to less than 2 hours (Fig. 3).

### The role of PKC in LPS stimulated RAW cell NO production

**Figure 4:**
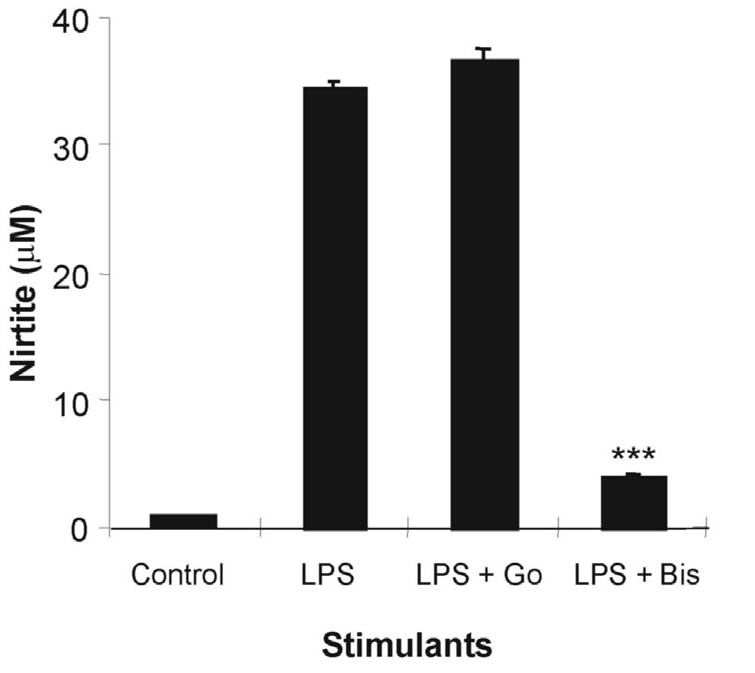
**Effect of PKC inhibitors on LPS induced NO production in RAW 264.7 cells**. Cells were treated with vehicle (control) or LPS (1 μg/ml) in the presence or absence of PKC inhibitors Go 6978 (Go, 10 μM) or bisindolylemaleimide (Bis, 20 μM) for 24 hours. The culture medium was then harvested and assayed for nitrite content by Greiss assay. The data show that only Bis was able to inhibit LPS-stimulated RAW cell NO production. Results are expressed mean ± SEM, ***p < 0.001, n = 9.

Effects on PKC isoforms degradation have been reported following PMA treatment at the concentration of PMA used in this study (50 ng/ml) [28]. The induction of NOS2 activity upon activation of PKC by LPS stimulation has also been demonstrated previously using a non-selective inhibitor of all PKC isoforms [16]. However, the role of specific PKC isoforms has remained unclear. The MALY peptides have been reported to mimic the PKC variable regions 1 and 2 (V1-2). These regions are necessary for binding PKC to the receptors for activated C kinase (RACK) and thereby prevent nuclear-cytoplasmic translocation of specific PKC isoforms [29]. The addition of a TAT sequence (GGGGYGRKKRRQRRR-GGGG) to the MALY peptides ensures that they are transported into the nucleus, an important facet for some PKC enzymes. Pre-treatment with these translocation inhibitor-peptides had no effect on NO production in these cells (Table 1). In addition, the PKC inhibitor Gö 6983 (Go, 10 μM) had no effect on LPS-stimulated NO production (Fig. 4).

**Table 1:** 

	Nitrite (μM); mean ± SEM						
	
	*Control*	*Concentration of peptide alone*		*1 μg/ml LPS with stated concentration of peptides below*			
		10 μM	100 μM	0 μM	1 μM	10 μM	100 μM
MALY O1	0.70	2.00	0.77	44.6	46.1	47.2	51.6
	± 0.2	± 1.6	± 0.3	± 2.7	± 4.2	± 5.1	± 2.0
MALY 1O	0.70	0.87	0.85	44.6	47.3	40.3	46.4
	± 0.2	± 0.4	± 0.3	± 3.3	± 0.8	± 1.9	± 0.9
MALY II	0.70	0.64	0.66	44.6	46.2	43.4	45.9
	± 0.2	± 0.3	± 0.3	± 2.8	± 0.9	± 2.9	± 0.5

RAW cells were stimulated with medium alone (control), LPS (1 μg/ml) or various concentrations of the MALY TAT-linked PKC translocation inhibitor peptides (MALYO1 = cPKC inhibitor, MALY1O = PKC specific, MALYII = scrambled PKC inhibitor peptide), with or without LPS for 24 hr. NO production was assessed by Greiss assay. Results represent mean ± SEM (n= 3) and are representative of two separate experiments.

**Figure 5:**
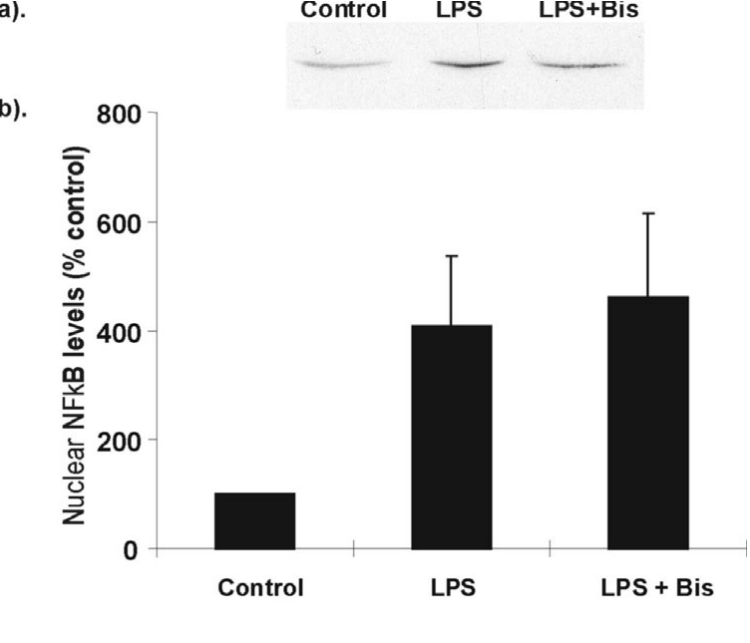
**Effect of bisindolylemaleimide (Bis) on LPS stimulated NF-κB activation in RAW 264.7 cells**. RAW cells were stimulated with LPS (1 μg/ml) in the presence or absence of Bis (20 μM) for 3 hr. Cells were harvested and nuclear NF-κB-p65 levels were assessed by Western blotting. (a) Representative Western blot of p65 expression. Equal amounts of nuclear proteins were loaded onto each lane. (b) Graphical representation of % increase in p65 nuclear localisation is shown in (a) above. Data is presented as mean ± SEM, n = 3 independent measurements.

In contrast, bisindolymalemide I (Bis, 20 μM) completely inhibited LPS (1 μg/ml) -stimulated NO production (Fig. 4). This was not due to an inhibitory effect on peak NF-κB p65 nuclear translocation as Bis (20 μM) had no effect on LPS-stimulated nuclear translocation at 3 hr (Fig. 5). This time point was chosen as it represented the time at which NF-κB p65 nuclear translocation was at its peak (Fig. 2). The results also contrast with the effect of PMA which returned p65 nuclear translocation to baseline within 2 hr.

### The effect of AG-490 on LPS induced RAW cell NO production

**Figure 6:**
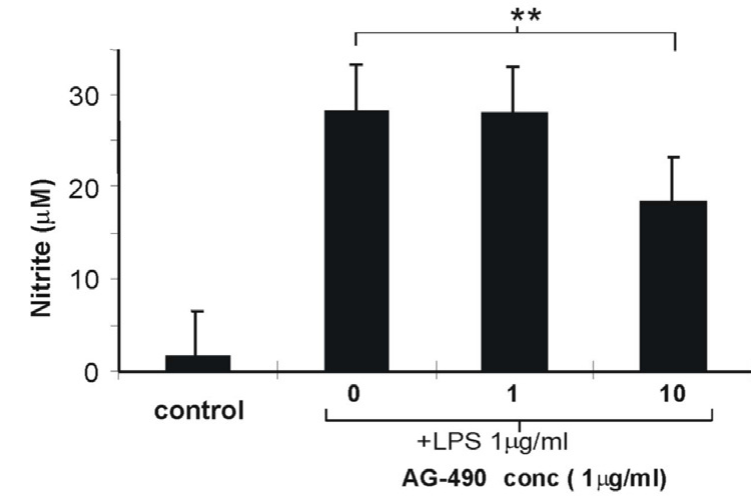
**Effect of JAK2 inhibitor on LPS-stimulated NO production in RAW 264.7 cells**. Concentration dependent effect of the JAK2 inhibitor AG-490 (0–10 μM) on LPS (1 μg/ml)-stimulated NO production measured at 24 hr. Cells were stimulated and the culture medium harvested and assayed for nitrite content by Greiss assay. The data show that AG-490 was able to inhibit LPS-stimulated NO production. Results for the effects of the inhibitor are expressed mean ± SEM, **p < 0.01 vs no inhibitor, n = 6.

AG-490, a potent inhibitor of JAK2, caused a concentration-dependent inhibition of LPS-stimulated NO production and this was significant at 10 μM (Fig. 6). JAK2 has been shown to be activated in response to a wide variety of stimuli [30] and AG-490 to block induced NOS2 expression in IL-1β/TNFα/IFNγ-stimulated human epithelial-like colon carcinoma DLD-1 cells [31] and in IFNγ/LPS stimulated RAW cells [32]. This present finding extends the previous studies and further indicates the involvement of JAK2 in NF-κB-induced LPS-stimulated NO production and NF-κB activation in RAW cells.

### The effect of SB 203580-induced inhibition of p38 on LPS induced RAW cell NO production

**Figure 7:**
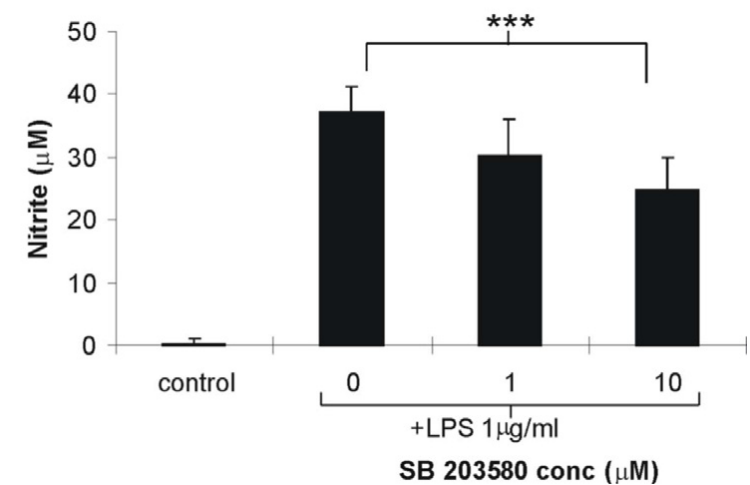
**Effect of SB203580 on LPS stimulated NO production**. Concentration dependent effect of the p38 MAPK inhibitor SB203580 (0–10 μM) on LPS (1 μg/ml)-stimulated NO production measured at 24 hours. Cells were stimulated and the culture medium harvested and assayed for nitrite content by Greiss assay. The data show that SB203580 was able to inhibit LPS-stimulated NO production. Results for the effects of the inhibitor are expressed mean ± SEM, ***p < 0.001 vs no inhibitor, n = 6.

p38 MAP kinase has previously been implicated in NF-κB activation [33] and in IFNγ/LPS stimulated NOS2 expression in RAW 264.7 cell, although this has not been reported for LPS-stimulated cells alone [34]. SB203580 inhibited LPS-stimulated NO production in a concentration-dependent manner with an IC_50 _of ~3 μM indicating selectivity of this effect. Maximal inhibition (~35%) was seen at 10 μM (Fig. 7).

## Discussion

This study investigated the effect of distinct kinase pathways on their ability to modulate NF-κB activation and thereby modify LPS stimulated NO production in RAW macrophages. Previous findings have been extended, with NF-κB-mediated NO production stimulated by LPS is shown to be multifactorial in nature, involving the co-ordinated activation of PKCε, JAK2 and p38 MAPK. The differential effects of the PKC inhibitors Go and Bis suggested that PKCε was involved in NO release.

Under unstimulated conditions, p65 is restricted to the cytoplasm by a set of inhibitory proteins and, upon stimulation, translocated to the nucleus. This stimulation can be modulated by phosphorylation of p65 at serine residues. The degree of activation by NF-κB is thus likely to result from a combination of p65 nuclear translocation and post-translational modifications of p65 [35].

It is evident from many studies that LPS-stimulated NO release from RAW macrophages is NF-κB dependent [36]. In the present study, although PMA enhanced the amount of p65 nuclear translocation, it also decreased the period over which LPS was able to maintain NF-κB nuclear translocation and this may be linked to the reduction in NO release.

Inhibition of PKCε by Bis had no effect on nuclear translocation in our study confirming a previous report in LPS-stimulated blood monocytes [37]. This data, in conjunction with the data showing a lack of effect of a TAT-linked MALY inhibitor, indicates that PKC is not involved in the nuclear translocation or DNA-binding of NF-kB. Thus, PKCε probably acts on nuclear NF-κB to either affect its nuclear retention or more likely to affect p65 transcriptional activity through a posttranslational modification event leading to differential recruitment or activation of transcriptional co-activators. Indeed, a PKC phosphorylation site exists on the p65 subunit and such phosphorylation is known to increase the transactivation potential of NF-κB without affecting its DNA binding or nuclear translocation [38,39]. Furthermore, the data presented here suggests that the effect of PMA on LPS-stimulated NO release is not through a PKC-mediated effect but that PMA induces additional pathways that regulate LPS-induced NF-κB activation and NO production. Thus, altered PKC activity may also impinge upon the NF-κB functional response either by affecting co-factor or histone phosphorylation [40].

A role for JAK2 in LPS and IFN stimulated NO production in RAW cells has been described previously [41]. However, JAK2 was hypothesised to work solely through STAT1 activation and be activated by IFNγ. There is currently growing evidence for cross talk between the JAK2 and the NF-κB signalling pathway [42] and also the JNK pathway indirectly through an effect on PI3K [43,44]. JAK2 has been demonstrated to phosphorylate IkB thereby facilitating NF-κB activation [14]. AG-490 has also been reported to inhibit LPS stimulated NF-κB activation and subsequent NOS2 induction in a skin dendritic cell line [45].

As with JAK2, the results from the present study suggest that the p38 MAPK protein (MAP) kinase is also involved in NF-κB activation [29,33] and therefore LPS-induced NO production. Previous reports have shown equivocal data as to the role of p38 MAPK in these events [46,47]. Although p38 MAPK does not appear to be involved in NO release induced by other agents in RAW cells [48]. The IC_50 _of inhibition of SB203580 indicates relative selectivity and further analysis of LPS-induced p38 MAPK activation or the use of more selective inhibitors may provide additional evidence for its role effects in these cells and for p38 inhibitors potential in the treatment of LPS activated disease.

**Figure 8:**
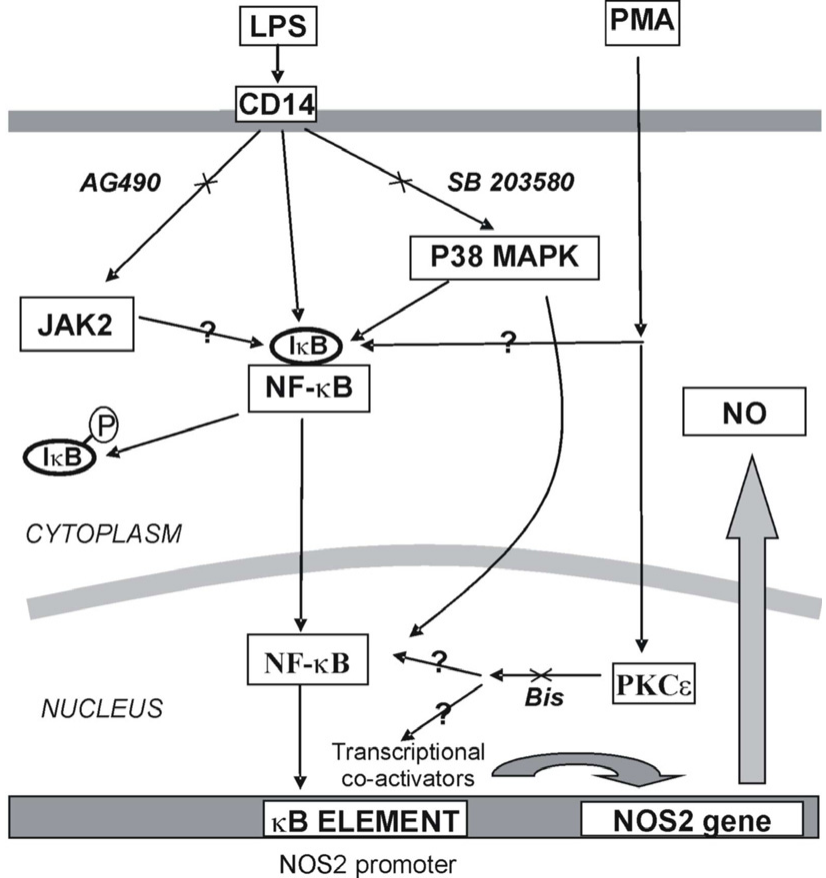
Diagram showing a pictorial representation of the conclusions from all experiments (? = areas of uncertainty).

In conclusion, the findings of the present study demonstrate the role of NF-κB in LPS stimulated NO production in RAW cells and indicate the importance of cross-talk with other kinase pathways, namely PKCε. Furthermore, the present findings further define the involvement of PKCε and JAK2 in inducing NO production, probably through their effects on NF-κB induced NOS2 expression. Figure 8 provides a pictorial summary of these findings. However, these conclusions have been drawn on the basis of use of well characterised inhibitors, rather then actual measurement of the activity of the target proteins which would provide further confirmation of this regulatory network. The work presented here further illustrates the complex network of signalling pathways involved in modulation of NF-κB-mediated gene transcription.

## Competing interests

The author(s) declare that they have no competing interests.

## Authors' contributions

EJ carried out the cell culture experiments, molecular studies and analysis and presentation of the results and the initial interpretation. IA provided the training and expertise for the molecular studies, jointly conceived the study, participated in its design and coordination and significantly contributed to the drafting of the manuscript. BA contributed expertise and critical knowledge of the molecular studies and redrafted and formatted the early draft of the manuscript. NP initiated the project, jointly conceived the study, raised the initial funding, provided training in cell culture techniques, supervised the work of EJ and produced the final drafts of the manuscript. All authors read and approved the final manuscript.
